# HIV/HBV coinfection: temporal trends and patient characteristics, Spain, 2002 to 2018

**DOI:** 10.2807/1560-7917.ES.2021.26.25.2000236

**Published:** 2021-06-24

**Authors:** Leire Pérez-Latorre, Juan Berenguer, Rafael Micán, Marta Montero, Carmen Cifuentes, Teresa Puig, José Sanz, Oscar L Ferrero, Belén De La Fuente, Carmen Rodríguez, Sergio Reus, José Hernández-Quero, Gabriel Gaspar, Laura Pérez-Martínez, Coral García, Luis Force, Sergio Veloso, Marta De Miguel, Inmaculada Jarrín, Juan González-García, the GeSIDA 8514 Study Group, The GeSIDA 8514 Study Group, The GeSIDA 8514 Study Group, C Fanciulli, P Miralles, JC López, F Parras, B Padilla, T Aldámiz-Echevarría, A Carrero, C Díez, F Tejerina, J Berenguer, V Hontañón, C Busca, A Delgado, F Arnalich, JR Arribas, JI Bernardino, R De Miguel, ML Martín-Carbonero, R Montejano, ML Montes, V Moreno, I Pérez-Valero, E Valencia, MJ Vivancos, S Moreno, A Moreno, JL Casado, MJ Pérez-Elías, C Quereda, L Domínguez, A Hernando, O Bisbal, M De Lagarde, M Matarranz, R Rubio, F Pulido, J Navarro, A Torrella, N Ramos, M Rodrigo, V Estrada, J Vergas, MJ Téllez, J Muñoz, M Gutiérrez, G Mateo, JM Guardiola, M Ibarguren, MP Carmona, F Rodríguez-Arrondo, MA Goenaga, H Azkune, MA Von Wichmann, JA Iribarren, T Brieva, A Camacho, IM Machuca, A Rivero-Juárez, A Rivero-Román, J Ruiz, E Nuño, R Palacios, J Santos, M Márquez, J Carmena, A Artero, L Morano, M Crespo, L García, S Otero, J Sanz, I Santos, J Moreno, P Arazo, C Armiñanzas, S Echevarría, M Gutiérrez-Cuadra, MC Fariñas, A Ferrer, MJ Galindo, M Tasias, S Cuellar, E Calabuig, M Blanes, J Fernández, J López-Aldeguer, M Salavert, P Domingo, J de Miguel, A Arranz, E Casas, OL Ferrero, S Ibarra, I López, M de la Peña, Z Zubero, J Baraia, J Muñoz, M Campoamor, MJ Tuya, C Rodríguez, T Puerta, M Raposo, M Vera, J Del Romero, S Reus, L Giner, E Merino, V Boix, D Torrús, I Portilla, M Pampliega, M Díez, I Egea, J Portilla, D Vinuesa, L Muñoz, L García, JA Oteo, C García, P Barrufet, J Peraire, C Viladés, M Vargas, A Castellano, F Vidal, M Velasco, L Moreno, R Hervás, JE Losa, J Vilaró, A Cano, A Alcaráz, A Muñoz, E Bernal, A Gimeno, C Montero, S Arponen, AJ Orti, E Chamarro, C Escrig, A Chocarro, R Teira, G Alonso, C Toledo, AI Peláez, G Lara, I Fernández, MC Esteban, E Gómez-Alfaro, R Silvariño, A Vegas, P Geijo, J Bisbe

**Affiliations:** 1 Hospital General Universitario Gregorio Marañón, Madrid, Spain; 2 Hospital Universitario La Paz, Madrid, Spain; 3 Hospital Universitario La Fe, Valencia, Spain; 4 Hospital Son Llàtzer, Son Ferriol, Spain; 5 Hospital Universitari Arnau de Vilanova, Lleida, Spain; 6 Hospital Príncipe de Asturias, Alcalá de Henares, Spain; 7 Hospital de Basurto, Bilbao, Spain; 8 Hospital de Cabueñes, Gijón, Spain; 9 Centro Sanitario Sandoval, Madrid, Spain; 10 Hospital General Universitario de Alicante, Alicante, Spain; 11 Hospital Clínico Universitario San Cecilio, Granada, Spain; 12 Hospital Universitario de Getafe, Getafe, Spain; 13 Hospital General de La Rioja, Logroño, Spain; 14 Hospital Universitario Virgen de las Nieves, Granada, Spain; 15 Hospital de Mataró, Mataró, Spain; 16 Hospital Universitari Joan XXIII, Tarragona, Spain; 17 Fundación SEIMC/GESIDA, Madrid, Spain; 18 Instituto de Salud Carlos III, Madrid, Spain; 19 The members of the GeSIDA 8514 Study Group have been listed under Investigators

**Keywords:** HIV/HBV coinfection, HBV therapy, hepatitis delta virus

## Abstract

**Background:**

Recent and reliable estimates on the prevalence of coinfection with human immunodeficiency virus (HIV) and hepatitis B virus (HBV) in Europe are lacking.

**Aim:**

Leveraged on a study designed to assess HIV/HCV coinfection prevalence, we assessed the prevalence of HIV/HBV coinfection in Spain in 2018 and compared the results with five similar studies performed since 2002.

**Methods:**

This cross-sectional prevalence study was carried out in 43 centres, and patients were selected using simple random sampling. The reference population comprised 40,322 patients and the sample size were 1,690 patients.

**Results:**

The prevalence of HIV/HBV coinfection in Spain at the end of 2018 was 3.2%. The prevalence in 2002, 2009, 2015, 2016 and 2017 was 4.9%, 3.4%, 3%, 3.9% and 3%, respectively. Among the HIV/HBV-coinfected patients identified in 2018, 16.7% had cirrhosis according to transient elastography and 26.3% tested positive for antibodies against hepatitis D virus. All HIV/HBV-coinfected patients were receiving drugs with activity against HBV, and 97% of those tested for HBV DNA had an HBV DNA load < 80 IU/mL.

**Conclusions:**

The prevalence of HIV/HBV coinfection in Spain remained stable at around 3% for a decade. Our data could facilitate the design of national programmes to control HBV infection and help identify areas of patient management that need improvement.

## Introduction

People living with human immunodeficiency virus (PLHIV) are potentially at high risk of being infected with hepatitis B virus (HBV), as both viruses share transmission routes. HIV/HBV coinfection has been associated with increased levels of HBV DNA, accelerated progression of liver disease and increased all-cause and liver-related mortality [1-4].

Globally, it is estimated that 10% of PLHIV are also coinfected with HBV, although rates of coinfection vary considerably between regions and risk groups, reflecting marked differences in risk factors and HBV immunisation coverage [1,5]. A recent systematic review concluded that the majority of studies on HBV prevalence and incidence among PLHIV in Europe were hampered by quality issues, the most common being whether the study population was representative of the source population [6]. Studies that provide estimates of HIV/HBV coinfection are necessary to facilitate the design of national and local programmes in order to meet the World Health Organization (WHO) goal of reducing new viral hepatitis infections by 90% and reducing deaths due to viral hepatitis by 10% by 2020 and by 65% by 2030 [7]. Estimating the burden of HIV/HBV coinfection is also relevant because some of the antiretroviral drugs to be approved in the near future, including injectable long-acting drugs, lack activity against HBV [8].

We leveraged a study on HIV/HCV coinfection prevalence to determine the prevalence of HIV/HBV coinfection in Spain in 2018, compare the results with similar studies performed in the previous 15 years and analyse patients’ characteristics and treatment.

## Methods

### Setting and design

The study was carried out by the Grupo de Estudio del SIDA (AIDS study group; GeSIDA) of the Sociedad Española de Enfermedades Infecciosas y Microbiologıa Clınica (Spanish Society of Infectious Diseases and Clinical Microbiology, SEIMC) between 1 October and 30 November 2018, based on a similar methodology to that used in five previous studies performed in 2002, 2009, 2015, 2016 and 2017 [9]. The present national prevalence study of HIV/hepatitis C virus (HCV) and HIV/HBV coinfections included 43 hospitals throughout Spain. The reference population comprised all PLHIV in active follow-up in the participating centres. Active follow-up was defined as at least one visit to the centre in the previous 12 months. The sample size was estimated to assess the prevalence of active HCV infection with a 95% confidence level, a design effect of 1.0 and an accuracy for the sample size of 1.25%. Considering that the prevalence of active HCV infection was 8.0% in the most recent survey conducted by GeSIDA in 2017 [10], we estimated that a sample of at least 1,733 patients was needed. Given that the prevalence of HIV/HBV coinfection was estimated to be 3% in 2017 [11], the accuracy for estimation of the prevalence of HIV/HBV coinfection with this sample size was 0.8%. The number of patients to be included at each centre was determined by proportional allocation, and patients were selected by simple random sampling.

### Data collection

We collected demographic data, HIV transmission category, the United States (US) Centers for Disease Control and Prevention (CDC) category that classifies patients with HIV according to clinical and immunological parameters [13], current CD4^+^ T-cell counts, current HIV RNA detectability, whether patients were on combination antiretroviral therapy (ART) and the regimen used. We also recorded the presence of HBV surface antigen (HBsAg), presence of HCV antibodies and, if applicable, presence of HCV RNA. In patients with HBsAg, information was also obtained about anti-HBV therapy, hepatitis D virus (HDV) antibodies, HBV DNA load and liver fibrosis. We used the cut-off values recommended by the European AIDS Clinical Society for transient elastography for the detection of advanced fibrosis and cirrhosis in patients with HIV/HBV coinfection [14]. All the information was entered into a shared database at each institution using an online electronic case report form.

### Data analysis

We carried out a descriptive analysis using frequency tables for categorical variables, mean and standard deviation (SD) for normally and median and interquartile range (IQR) for non-normally distributed continuous variables. We used the chi-square test of independence to detect significant differences in categorical variables and Student’s t-test and the Mann–Whitney test, respectively, to assess differences between normally and non-normally distributed continuous variables. All statistical analyses were performed using Stata, version 14.0 (StataCorp, College Station, US).

### Ethical statement

The planning conduct and reporting of studies was in line with the Declaration of Helsinki, as revised in 2013 [12]. The Institutional Ethics Committee of Hospital General Universitario Gregorio Marañón approved the study (Reference: SEI-VIII-2015-01) and waived the requirement for written informed consent because the study was based on anonymous routine clinical data intended for scientific publication.

## Results

A total of 43 centres participated in the study. The reference population was 40,650 PLHIV, and the sample size was 1,733 patients, of whom 54 (3.1%) tested positive for HBsAg, 1,619 (93.4%) tested negative for HBsAg and 60 (3.5%) had unknown results. Therefore, the prevalence of HBV coinfection in PLHIV in Spain in 2018 was 3.2% (95% confidence interval (CI): 2.4–4.2). The prevalence of HIV/HBV coinfection in all the studies carried out by GeSIDA since 2002 is shown in the Figure. The prevalence of HIV/HBV coinfection in Spain was highest in 2002 (4.9%) after which it has remained stable, varying from 3.0% to 3.9% between 2009 and 2018. Minor variations in the number of participating centres occurred along the years; however, the vast majority of hospitals remained on the same level.

**Figure:**
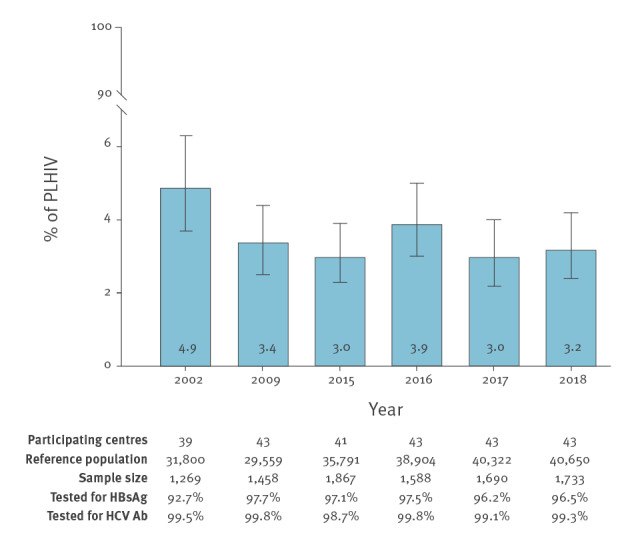
Prevalence of hepatitis B virus infection (HBsAg-positive) among people living with HIV, Spain, 2002–2018 (n = 9,605)

Ab: antibodies; HBsAg: hepatitis B virus surface antigen; HCV: hepatitis C virus; PLHIV: people living with human immunodeficiency virus.

The characteristics of the 1,733 PLHIV included in the 2018 survey are summarised in the Table.

**Table:** Baseline characteristics of the patients included in the study on the prevalence of HIV/HBV coinfection, Spain, October−November 2018 (n = 1,733)

Characteristic	HBsAg						P^a^	Total	
	Unknown		Positive		Negative			n	%
	n	%	n	%	n	%			
Total	60	3.5	54	3.1	1,619	93.4	NA	1,733	100
Male sex	44	73.3	44	81.5	1,202	74.2	0.230	1,290	74.4
Mean age in years (SD)	51 (10)		50 (11)		49 (11)		0.367	49 (11)	
HIV transmission category									
Men who have sex with men	7	11.7	21	38.9	603	37.2	0.174	631	36.4
Injection drug use	19	31.7	21	38.9	455	28.1		495	28.6
Heterosexual	15	25.0	8	14.8	440	27.2		463	26.7
Contaminated blood products	0	0	1	1.9	10	0.6		11	0.6
Mother-to-child transmission	0	0	1	1.9	12	0.7		13	0.8
Other	19	31.7	2	3.7	99	6.1		120	6.9
CDC clinical category C [13]									
No	45	75.0	38	70.4	1,174	72.5	0.939	1,257	72.5
Yes	13	21.7	15	27.8	419	25.9		447	25.8
Unknown	2	3.3	1	1.8	26	1.6		29	1.7
HCV antibodies									
Negative	26	43.3	32	59.3	1,085	67	0.445	1,143	65.9
Positive	26	43.3	22	40.7	530	32.7		578	33.4
Unknown	8	13.3	0	0	4	0.2		12	0.7
HCV RNA (among the HCV antibody-positive)									
Positive	4	15.4	3	13.6	57	10.8	< 0.001	64	11.1
Negative after anti-HCV therapy	20	76.9	7	31.8	382	72.1		409	70.8
Negative spontaneous clearance	2	7.7	12	54.5	90	17.0		104	18.0
Unknown	0	0	0	0	1	0.2		1	0.2
ART									
Yes	60	100	52	96.3	1,587	98.0	0.601	1,699	98
No	0	0	2	3.7	30	1.9		32	1.8
Unknown	0	0	0	0	2	0.1		2	0.1
Type of cART regimen (among those on ART)									
2 NRTI + 1 INSTI	23	38.3	24	46.1	701	44.2	0.272	748	44.0
2 NRTI + 1 NNRTI	12	20.0	17	32.7	359	22.6		388	22.8
2 NRTI + 1 PI	5	8.3	5	9.6	194	12.2		204	12.0
PI-based dual therapy	6	10.0	0	0	95	6.0		101	5.9
PI-based monotherapy	2	3.3	2	3.8	54	3.4		58	3.4
Other regimens	12	20.0	4	7.7	184	11.6		200	11.8
HIV RNA copies/mL									
All patients									
< 50	52	86.7	50	92.6	1,485	91.7	0.697	1,587	91.6
50–200	4	6.7	1	1.9	70	4.3		75	4.3
> 200	4	6.7	3	5.6	59	3.6		66	3.8
Unknown	0	0	0	0	5	0.3		5	0.3
Patients on cART									
< 50	52	86.7	50	96.1	1,478	93.1	0.819	1,580	93.0
50–200	4	6.7	1	1.9	69	4.3		74	4.4
> 200	4	6.7	1	1.9	35	2.2		40	2.3
Unknown	0	0	0	0	5	0.3		5	0.3
CD4^+^ T-cells/µL, median (IQR)									
All patients	598 (410–951)		637 (343–836)		698 (480–919)		0.031	695 (471–915)	
Patients on cART	598 (410–951)		648 (371–842)		704 (480–921)		0.077	699 (473–919)	

ART: antiretroviral treatment; cART: combination antiretroviral therapy; CDC: Centers for Disease Control and Prevention; HBsAg: hepatitis B surface antigen; HCV: hepatitis C virus; HIV: human immunodeficiency virus; INSTI: integrase strand transfer inhibitor; IQR: interquartile range; NA: not applicable; NRTI: nucleoside reverse transcriptase inhibitor; NNRTI: non-nucleoside reverse transcriptase inhibitor; PI: protease inhibitor; SD: standard deviation. Ab: antibodies; HBsAg: hepatitis B virus surface antigen; HCV: hepatitis C virus.

^a^ p values for the comparisons between HBsAg-positive patients and HBsAg-negative patients.

In brief, 74.4% of patients were male, median age was 49 years (SD: 10) and the most frequent self-reported HIV transmission categories were men who have sex with men (MSM) (36.4%), injection drug use (IDU) (28.6%) and heterosexual relations (26.7%). Prior AIDS-defining conditions (CDC clinical category C) were reported by 25.8%, and 98.0% were receiving ART. The median CD4^+^ T-cell count was 695 (IQR: 471–915) cells/µL and 93% of patients on ART had an HIV viral load lower than 50 copies/mL. No statistically significant differences were found between HBsAg-positive patients and HBsAg-negative patients in baseline characteristics except for a higher percentage of spontaneous clearance of HCV among the former (54.5% vs 17.0%; p < 0.001). Information about HDV antibodies was available from 38 of 54 patients with HIV/HBV coinfection, 10 of whom had positive results. Transient elastography was performed during the previous 12 months in 30 patients with HIV/HBV coinfection: 10 patients had a value ≥ 7.6 kPa, which is suggestive of advanced liver fibrosis, and five patients had a value ≥ 9.4 kPa, which is compatible with liver cirrhosis. All 54 HIV/HBV-coinfected patients were receiving antiretroviral drugs with activity against HBV, as follows: 45 received tenofovir (disoproxil fumarate or alafenamide), 36 emtricitabine, 13 lamivudine, and two received entecavir. HBV DNA had been determined in the previous 12 months in 33 patients, of whom 32 had an HBV DNA load < 80 IU/mL and one an HBV DNA load between 80 and 2,000 IU/mL.

Among the 1,733 PLHIV included in the 2018 survey, HCV serology was known for 1,721 patients (99.3%), 578 of whom were HCV antibody-positive. Of the 578 patients with HCV antibodies, 409 were HCV RNA-negative following sustained viral response to anti-HCV therapy, 64 patients were HCV RNA-positive, 104 had cleared HCV RNA spontaneously, and HCV RNA status was unknown for one patient. The prevalence of HCV antibodies was thus 33.6%, and the prevalence of active HCV infection (HCV RNA positivity) was 3.7%. Self-reported HIV transmission categories among 64 patients with active HCV infection were IDU (n = 50), MSM (n = 7), heterosexual relations (n = 2), and other/unknown (n = 5). Active infection by HBV and HCV was identified in three PLHIV in the 2018 survey.

## Discussion

The prevalence of HBV coinfection among PLHIV in Spain at the end of 2018 was 3.2%, almost five times higher than in the general population in Spain, which was estimated to be 0.6–0.7% in recent surveys [15,16], but three times lower than the 10.4% prevalence found among PLHIV across the European Union and European Economic Area (EU/EEA) [6].

The various studies carried out by GeSIDA show that the prevalence of HIV/HBV coinfection in Spain was highest in 2002 (4.9%) and has since remained stable, varying from 3.0% to 3.9% between 2009 and 2018. These findings contrast considerably with the sharp decrease in the prevalence of active HCV infection in the country from 54.0% in 2002 to 3.7% in 2018 [17], indicating that HBV is likely to be the leading cause of chronic viral hepatitis among PLHIV in Spain at the time of writing. Coinfection by HBV and HCV was a highly uncommon event, recognised in less than 0.2% of PLHIV in the 2018 survey.

The persistent figures of HIV/HBV coinfection are somewhat surprising given that HBV vaccination coverage has increased steadily in Spain since its introduction in high-risk groups in 1982 [18] and given that HBV-active antiretroviral drugs with a protective effect against primary HBV infection are used frequently [19,20]. However, it must be taken into account that the proportion of PLHIV in Spain who were born in areas with a high prevalence of HBV infection, such as Latin America, Eastern Europe and Africa, has increased from 5.1% in 2002 to 13.7% in 2017 [21].

HDV serology was determined in approximately two thirds of HIV/HBV-coinfected patients included in this survey, 26% of whom tested positive. This percentage is 6.5 times higher than the 4% prevalence of HDV antibodies among HBV-infected patients without HIV infection in Spain [22] and 1.8 times higher than the 14.5% prevalence of HDV antibodies among HIV/HBV-coinfected patients in EuroSida [23]. Transient elastography was performed in the preceding year in slightly more than half of all patients with HIV/HBV coinfection, of whom 16.7% met the criteria for cirrhosis. This percentage is higher than the prevalence of cirrhosis in PLHIV with active HCV infection in Spain during the same year (10.9%) [17]. All HIV/HBV-coinfected patients in this survey were receiving drugs with activity against HBV, particularly tenofovir, and HBV viral load was fully suppressed in almost all of those tested for HBV DNA during the preceding 12 months.

As in other reports with HIV-infected and not HIV-infected persons, we found an association between HBV infection and spontaneous clearance of HCV [24-26]. Patients infected with both HBV and HCV may show a broad spectrum of virological profiles, and different viral dominance patterns have been documented [27]. In most cases, HCV is dominant and suppresses HBV replication; however, HBV can inhibit HCV replication. This is particularly true of HBV superinfection in which interferon-γ, interferon-α and tumour necrosis factor-α, which are released by host inflammatory cells in response to superimposed HBV infection, have been found to inhibit replication of HCV [28].

Our study is limited by the fact that we did not assess HBV vaccination coverage and effectiveness or occult HBV infection. In addition, the small sample size of patients with HIV/HBV coinfection is a limitation to analysing other potential differences between patients with and patients without HBV as well as associations between HDV seropositivity and any other potential risk factor. However, we report robust and accurate epidemiological data on the prevalence of HIV/HBV coinfection in Spain and on patient characteristics.

## Conclusion

The prevalence of HIV/HBV coinfection in Spain at the end of 2018 was 3.2%, which does not differ significantly from percentages reported in studies performed over the past 15 years. All HIV/HBV-coinfected patients were on anti-HBV drugs, and HBV DNA was fully suppressed in most cases. Liver cirrhosis and HDV infection were identified as common problems among HIV/HBV-coinfected patients. This information could facilitate the design of national programmes for control of HBV infection among PLHIV and help to identify areas of patient management that need improvement.
